# Anti-Complement, Anti-Oxidative, and Anti-Inflammatory Activities of the Ethanol Extract of *Tamarix chinensis* Lour.

**DOI:** 10.3390/plants15142199

**Published:** 2026-07-18

**Authors:** Muqing Wang, Min Cai, Xin Huang, Yu Liu, Congyu Wu, Yuan Gao, Yun Qi

**Affiliations:** State Key Laboratory of Bioactive Substance and Function of Natural Medicines, Institute of Medicinal Plant Development, Chinese Academy of Medical Sciences & Peking Union Medical College, Beijing 100193, China; 18353798604@163.com (M.W.); caiminsdu@163.com (M.C.); hx021124@163.com (X.H.); liuyu9985@126.com (Y.L.); wucongyu2021@163.com (C.W.)

**Keywords:** *Tamarix chinensis* Lour. (*T. chinensis*), anti-complement, anti-inflammation, anti-oxidative, NF-κB, AP-1

## Abstract

*Tamarix chinensis* Lour. (*T. chinensis*) is a traditional herb with functions for releasing the exterior to promote the eruption of rashes, among other ailments. However, these function-related pharmacological effects, such as anti-complement, anti-oxidative, and anti-inflammatory, remain unclear. This study aims to reveal the aforementioned effects and the molecular mechanisms of the ethanol extract of *T. chinensis* (TCE). Our results demonstrated that TCE inhibited classical- and lectin-mediated complement activation, reduced intracellular ROS via NADPH oxidase inhibition, and directly scavenged DPPH radicals and superoxide anions. By using lipopolysaccharide (LPS)-stimulated macrophages, along with LPS-induced acute lung injury (ALI) and endotoxemia mice, the anti-inflammatory activity and the underlying molecular mechanisms of TCE were deeply investigated. In LPS-activated macrophages, it suppressed iNOS, CCL2, IL-6 and IL-1β transcriptionally and translationally. Mechanistically, TCE inhibited NF-κB signaling by blocking IκBα phosphorylation and p65 nuclear translocation, as well as suppresses AP-1 signaling by reducing ERK and JNK phosphorylation. In vivo, TCE lowered serum multiple pro-inflammatory cytokines of endotoxemic mice and alleviated lung injury of ALI mice. Collectively, our results demonstrated that TCE possesses anti-complement and anti-oxidative activities and exerts anti-inflammatory effects through inhibiting NF-κB and AP-1 signaling. These findings provide scientific evidence for supporting the traditional use of *T. chinensis*.

## 1. Introduction

Inflammation is the body’s immediate response to damage caused by pathogens or harmful stimuli such as chemicals or physical injury to tissues and cells [1]. A controlled inflammatory response is a key self-defense mechanism, while excessive inflammation may cause major tissue damage and trigger inflammatory diseases [2]. As the sentinels of the immune system, macrophages are the major effector cells that respond to invading microorganisms during infectious inflammation. Lipopolysaccharide (LPS), a major pathogen-associated molecular pattern (PAMP) expressed on Gram-negative bacteria, can be recognized and bound by Toll-like receptor 4 (TLR4) located in the macrophage membrane [3,4]. After binding to LPS, TLR4 dimerizes and changes conformation, leading to the recruitment of myeloid differentiation primary response 88 (MyD88), and thus, subsequently activates two classical inflammation-related transcription factors: nuclear factor-κB (NF-κB) and activator protein-1 (AP-1) [5,6]. In the resting cells, NF-κB dimers (p50/p65) are sequestered in the cytoplasm as an inactive form due to linkage to its inhibitor, i.e., inhibitor of κB α (IκBα). Upon LPS stimulation, activated IκB kinase β (IKKβ) rapidly phosphorylates IκBα, triggering the ubiquitination and degradation of IκBα. As a result, the liberated NF-κB (p65/p50) translocates into the nucleus and initiates the expression of target genes related to the inflammatory response [7]. AP-1 stands as another pivotal nuclear transcription factor, which is regulated by the phosphorylation of the mitogen-activated protein kinases (MAPKs) family, including c-jun N-terminal kinase (JNK), extracellular signal-regulated kinase (ERK), and p38 mitogen-activated protein kinase (p38), which also exhibit a notable role in the inflammatory process [8]. These two pathways, when activated, trigger the transcription of downstream inflammation-related genes, including inducible nitric oxide synthase (iNOS), interleukin-1β (IL-1β), and interleukin-6 (IL-6), as well as c-c motif chemokine ligand 2 (CCL2) [9]. Thus, inhibition of NF-κB and/or AP-1 signaling is considered a beneficial therapeutic strategy for treating inflammatory diseases.

An imbalance between oxidant production and anti-oxidant defenses gives rise to oxidative stress, which not only causes cellular damage but is a key factor in many disorders, such as inflammatory diseases [10]. Under physiological conditions, reactive oxygen species (ROS) produced by phagocytic cells exert direct microbicidal effects by compromising microbial membrane integrity, facilitating efficient pathogen elimination by the host [11]. Furthermore, ROS play an essential role in maintaining cellular homeostasis by participating in key processes such as intracellular signaling, differentiation, and apoptosis [12], and can be efficiently eliminated by the anti-oxidant defense mechanisms consisting of enzymatic scavengers and non-enzymatic molecules, a system that ensures ROS signals are controlled [13]. Excessive ROS can result from either overproduction or impaired scavenging; however, this accumulation of ROS can induce oxidative damage to lipid peroxidation of membranes, protein carbonylation, and DNA strand breaks, resulting in multiple pathophysiological processes including inflammation, and ultimately leading to cell damage and death [14]. Therefore, modulation of oxidative stress also represents an important strategy for the treatment of various disorders, including inflammation.

**Figure 1:**
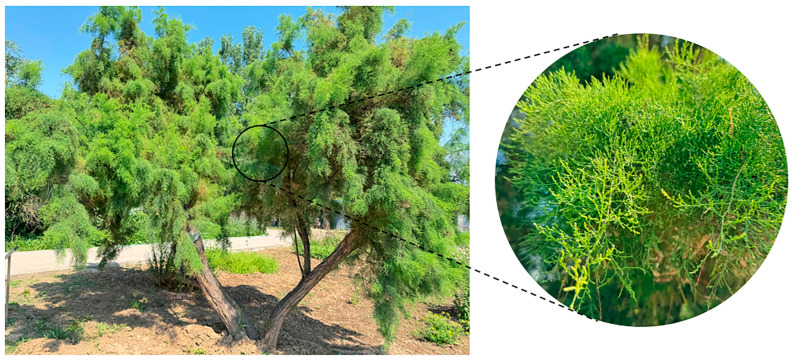
The original plant and the delicate branches and leaves of *Tamarix chinensis* Lour.

The genus Tamarix, belonging to the Tamaricaceae family, is a long-lived halophyte plant comprising approximately 104 species [15]. These species are characterized by needle-like leaves and are primarily distributed in hot, arid regions worldwide. Many species in this genus are used as traditional medicines for the treatment of various diseases, especially in Asian and African countries [16]. For this reason, pharmacological studies of this genus have drawn considerable attention, and their crude extracts or phytochemicals have been revealed to possess a broad spectrum of biological activities, including anti-inflammation, anti-Alzheimer, anti-cancer, anti-diabetes, and anti-microbial activities [16]. Among all Tamarix plants, *Tamarix chinensis* Lour. (*T. chinensis*) (Figure 1) is the only medicinal species in China and was first recorded in the “Supplement to Materia Medica (本草拾遗)”. It can release the exterior, promote the eruption of rashes, dispel wind-dampness, clear lung heat, and exert diuretic effects. The leaves and twigs of *T. chinensis*, known as “Xiheliu”, are also officially listed in the Chinese pharmacopoeia [17]. Clinically, it has mainly been used for the treatment of inflammation-related diseases, such as measles, pneumonia, and rheumatoid arthritis [18], in which the complement system is deeply involved and critically contributes to the pathology [19,20,21,22,23,24]. Phytochemical analysis reveals that Xiheliu contains various constituents, including flavonoids, terpenoids, organic acids, steroids, and volatile oil [25]. Its macromolecules, such as crude polysaccharides (MBAP90) and some homogeneous polysaccharides, show anti-complement and anti-oxidant activities in vitro [26]. Further studies found that MBAP90 and its homogeneous polysaccharides could alleviate virus-induced acute lung injury [18,27]. Given that Xiheliu is traditionally taken with alcohol or applied externally, we infer that small-molecule components in *T. chinensis* are more likely to be its active ingredient. The present study investigated the anti-complement, anti-oxidative, and anti-inflammatory properties of the ethanol extract of *T. chinensis* (TCE).

## 2. Results

### 2.1. High-Performance Liquid Chromatography (HPLC) Analysis of TCE

**Figure 2:**
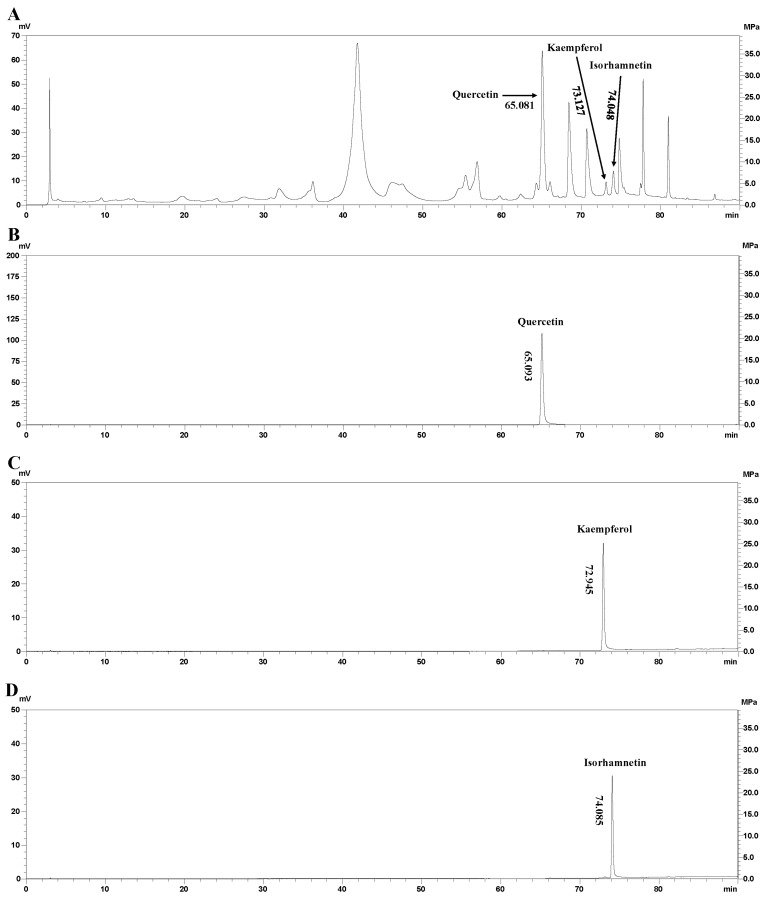
HPLC chromatograms of TCE and reference substances. (**A**) The fingerprint of TCE. (**B**–**D**) The HPLC chromatograms of three reference substances: quercetin (CAS^#^117-39-5) (**B**), kaempferol (CAS^#^520-18-3) (**C**), and isorhamnetin (CAS^#^480-19-3) (**D**).

The fingerprint of the TCE is presented in Figure 2A. Three peaks were successfully resolved, which was achieved by comparing their retention times with those of reference substances. The peaks corresponded to quercetin, kaempferol, and isorhamnetin, respectively (Figure 2B–D). The external standard calibration curves of three reference substances were created to quantify three identified components in TCE. Their contents in the TCE were 0.239% (quercetin), 0.033% (kaempferol), and 0.080% (isorhamnetin), respectively.

### 2.2. Anti-Complement Activity of TCE

**Figure 3:**
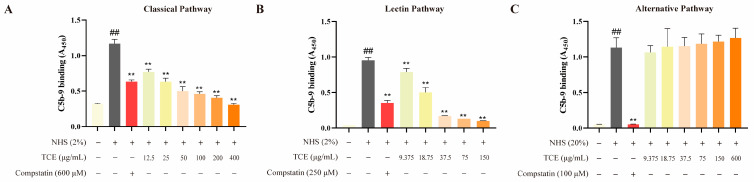
Anti-complement activity of TCE (*n* = 3). (**A**) Effect of TCE on classical pathway activation induced by IgM. (**B**) Effect of TCE on lectin pathway activation induced by mannan. (**C**) Effect of TCE on alternative pathway activation induced by zymosan A. Compstatin was used as a positive control. ^##^ *p* < 0.01 vs. negative control group, and ** *p* < 0.01 vs. model control group.

Complement activation occurs via three pathways: classical, lectin, and alternative pathways [28]. To evaluate the anti-complement activity of TCE, we used an ELISA-based system that allowed for the analysis of each pathway without cross-interaction [29]. Through coating specific activators, three complement pathways could be selectively initiated. Normal human serum (NHS) was added as a source of complement components [30]. As illustrated in Figure 3, compared with the negative normal control, all three activators significantly enhanced C5b-9 deposition in the model control group, and the positive control demonstrated varying degrees of inhibition on the generation of C5b-9 in NHS relative to the model control. Among the three pathways of complement activation, TCE specifically inhibited complement activation initiated by the classical and lectin pathways (Figure 3A,B), with half-maximal inhibitory concentration (IC_50_) values of 13.95 μg/mL and 18.31 μg/mL, respectively. Nevertheless, TCE failed to decrease C5b-9 deposition in the alternative pathway, even at concentrations up to 600 μg/mL (Figure 3C).

### 2.3. Anti-Oxidative Activity of TCE

#### 2.3.1. TCE Exerts Anti-Oxidative Activity in a Cell-Free System

**Figure 4:**
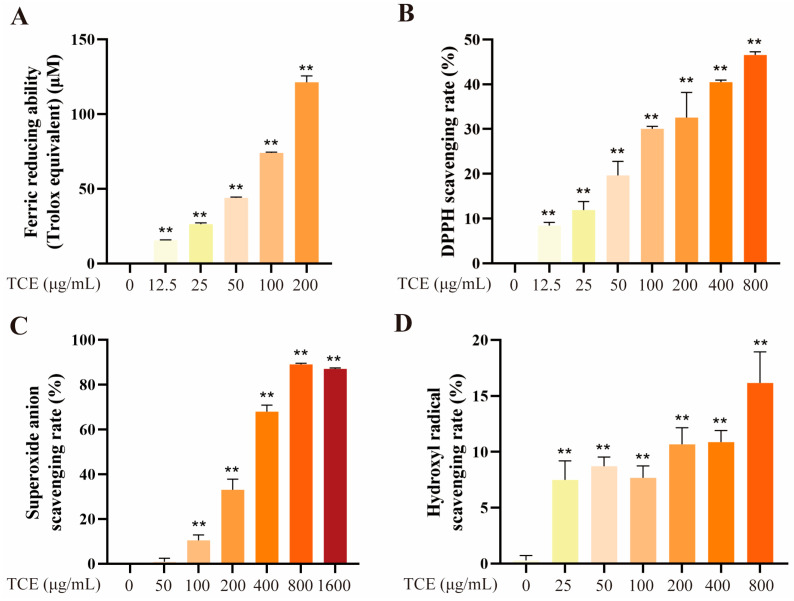
TCE exerts anti-oxidative activity in cell-free systems (*n* = 3). (**A**) Ferric reducing ability. (**B**) DPPH radical scavenging activity. (**C**) Superoxide anion scavenging activity. (**D**) Hydroxyl radical scavenging activity. Ferric reducing ability was expressed as Trolox (μM) equivalent anti-oxidant capacity.And ** *p* < 0.01 vs. 0 μg/mL group.

The cell-free evaluation of TCE focused on the following parameters: ferric reducing potential, as well as the capacity to scavenge 2,2-Diphenyl-1-picrylhydrazyl (DPPH) radicals, superoxide anions, and hydroxyl radicals. The ferric reducing ability of TCE was expressed as the Trolox equivalent concentration. The obtained result showed that 100 μg/mL of TCE was equivalent to about 74 μM of Trolox (Figure 4A). Moreover, TCE exhibited significant scavenging activity against both DPPH radicals and superoxide anions (Figure 4B,C). By comparison, when the concentration reached 800 μg/mL, TCE exhibited a hydroxyl radical scavenging rate of 16.16%, indicating limited hydroxyl radical scavenging activity under the experimental conditions (Figure 4D).

#### 2.3.2. TCE Exerts Anti-Oxidative Activity in the Cell System

**Figure 5:**
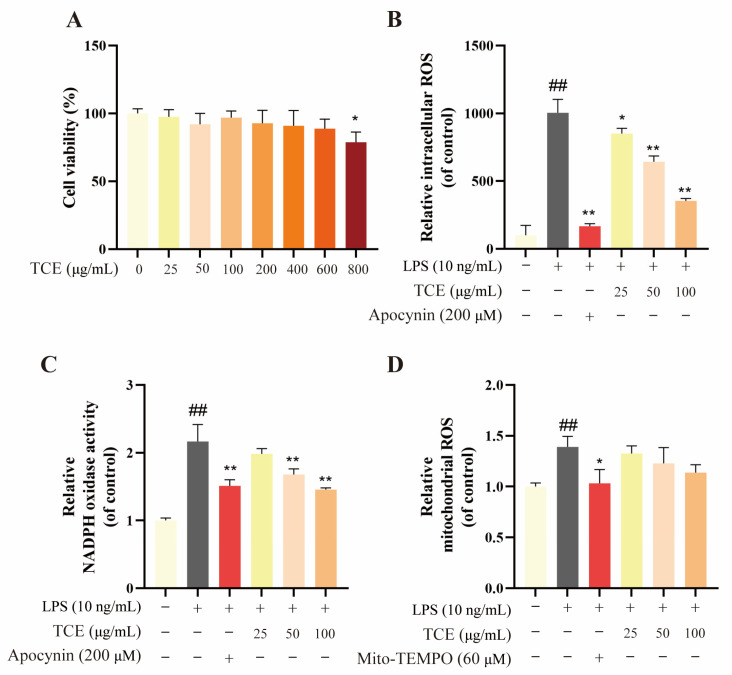
TCE exerts anti-oxidative activity in the cell system (*n* = 3). (**A**) Effect of TCE on the viability of RAW264.7 cells. (**B**) Effect of TCE on intracellular ROS. (**C**) Effect of TCE on intracellular NADPH oxidase activity. (**D**) Effect of TCE on mitochondrial ROS. Apocynin and Mito-TEMPO were used as positive controls. ^##^ *p* < 0.01 vs. normal control group; * *p* < 0.05 and ** *p* < 0.01 vs. LPS alone group.

To rule out the non-specific inhibitory action caused by cytotoxicity, we first determined the cytotoxic effect of TCE on the murine macrophage cell line (RAW264.7). The result demonstrated that TCE is non-toxic at concentrations up to 600 μg/mL compared with the negative control (Figure 5A). Thus, the following in vitro experiments were performed at non-toxic concentrations of TCE (25, 50, and 100 μg/mL). The data indicated that LPS treatment led to a robust increase in ROS production, while TCE could markedly reduce LPS-induced ROS accumulation (Figure 5B). Given the major contribution of nicotinamide adenine dinucleotide phosphate (NADPH) oxidase to intracellular ROS generation, we next determined how TCE affects NADPH oxidase activity. As demonstrated in Figure 5C, TCE indeed significantly inhibited LPS-induced NADPH oxidase activation; meanwhile TCE had no obvious effect on mitochondrial ROS (mtROS) production at the non-toxic concentrations (Figure 5D). These findings suggested that TCE decreased intracellular ROS via inhibition of NADPH oxidase activity; however, it did not affect mtROS production.

### 2.4. Anti-Inflammatory Activities of TCE In Vitro

#### 2.4.1. TCE Decreases Supernatant NO in LPS-Activated Macrophages

**Figure 6:**
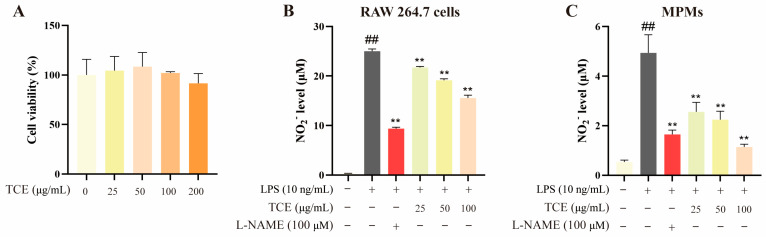
Effects of TCE on the cell viability and LPS-induced NO production in macrophages (*n* = 3). (**A**) Effects of TCE on the viability of MPMs. (**B**) Effects of TCE on supernatant NO in LPS-activated RAW264.7 cells. (**C**) Effects of TCE on supernatant NO in LPS-activated MPMs. ^##^ *p* < 0.01 vs. negative control (vehicle); ** *p* < 0.01 vs. LPS alone.

In addition to assessing the cytotoxic effect of TCE on the RAW264.7 cell line, we also determined whether TCE affects the viability of mouse peritoneal macrophage (MPM) cells. It was found that TCE is non-toxic at concentrations up to 200 μg/mL compared with the negative control (Figure 6A). Thus, the following in vitro assays were performed at non-toxic concentrations of TCE (25, 50, and 100 μg/mL). The effect of TCE on nitric oxide (NO), a micromolecule that can aggravate inflammation via oxidative damage to healthy cells and tissues [31], was first investigated. As shown in Figure 6B,C, LPS significantly increased NO production in two types of macrophages, while TCE markedly decreased supernatant NO in a concentration-dependent manner.

#### 2.4.2. TCE Inhibits iNOS Expression Rather than Its Activity in LPS-Activated Macrophages

**Figure 7:**
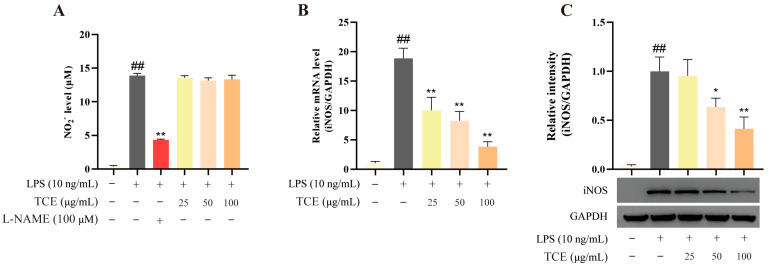
Effects of TCE on iNOS in LPS-stimulated RAW264.7 cells (*n* = 3). (**A**) Effect of TCE on iNOS activity in LPS-stimulated RAW264.7 cells. (**B**) Effect of TCE on the mRNA level of iNOS in LPS-stimulated RAW264.7 cells. (**C**) Effect of TCE on the protein level of iNOS in LPS-stimulated RAW264.7 cells. ^##^ *p* < 0.01 vs. negative control; * *p* < 0.05 and ** *p* < 0.01 vs. LPS alone.

In macrophages, LPS stimulation induces high iNOS expression, which is responsible for prolonged production of larger amounts of NO [32]. Any alteration in either the activity or the expression level of iNOS leads to a corresponding change in NO production. To assess the impact of TCE on iNOS activity, RAW264.7 cells were exposed to LPS for 12 h, and then the culture medium was removed. In this context, the amount of iNOS was fixed and the NO production only depended on the activity of iNOS. Unlike L-NAME (an iNOS inhibitor), TCE did not affect iNOS activity (Figure 7A). Next, the effect of TCE on iNOS expression was evaluated by RT-*q*PCR and Western blot. The results demonstrated that TCE decreased iNOS transcription and translation in a concentration-dependent manner (Figure 7B,C). These results suggest that TCE reduces NO level primarily by suppressing iNOS expression, not by directly inhibiting its activity.

#### 2.4.3. TCE Decreases the Production of IL-6, CCL2 and IL-1β in LPS-Activated Macrophages

**Figure 8:**
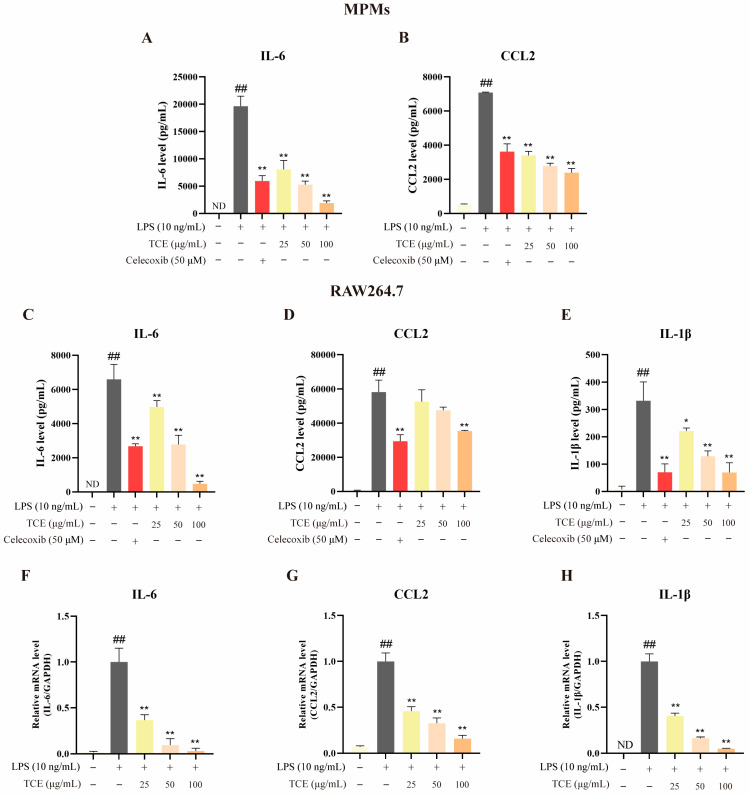
Effects of TCE on IL-6, CCL2 and IL-1β in LPS-primed macrophages (*n* = 3). (**A**,**B**) Effects of TCE on supernatant IL-6 and CCL2 in LPS-activated MPMs. (**C**,**D**) Effects of TCE on supernatant IL-6 and CCL2 in LPS-primed RAW264.7 cells. (**E**) Effect of TCE on intracellular pro-IL-1β in the cell lysates of LPS-primed RAW264.7 cells. (**F**–**H**) Effects of TCE on the mRNA levels of IL-6, CCL2 and IL-1β in LPS-primed RAW264.7 cells. ^##^ *p* < 0.01 vs. negative control; * *p* < 0.05 and ** *p* < 0.01 vs. LPS alone. ND, not detected.

In response to LPS stimulation, macrophages can also produce other pro-inflammatory cytokines, including IL-6, CCL2, and IL-1β. The effects of TCE on these three cytokines in LPS-activated macrophages were investigated next. The data indicated that administration of LPS upregulated the supernatant levels of IL-6 and CCL2. Pretreatment of TCE significantly downregulated these levels in a concentration-dependent manner in both MPMs and RAW264.7 cells (Figure 8A–D). Since IL-1β is not secreted via the conventional ER-Golgi pathway, RAW264.7 cells primed with LPS alone failed to secrete detectable IL-1β into the supernatant [33]. Therefore, intracellular pro-IL-1β was measured. The result showed that LPS significantly induced pro-IL-1β production, whereas TCE markedly decreased pro-IL-1β (Figure 8E). At the transcriptional level, TCE also potently suppressed LPS-induced IL-6, CCL2 and IL-1β mRNA expressions (Figure 8F–H).

#### 2.4.4. TCE Inhibits NF-κB Activation in LPS-Activated Macrophages

**Figure 9:**
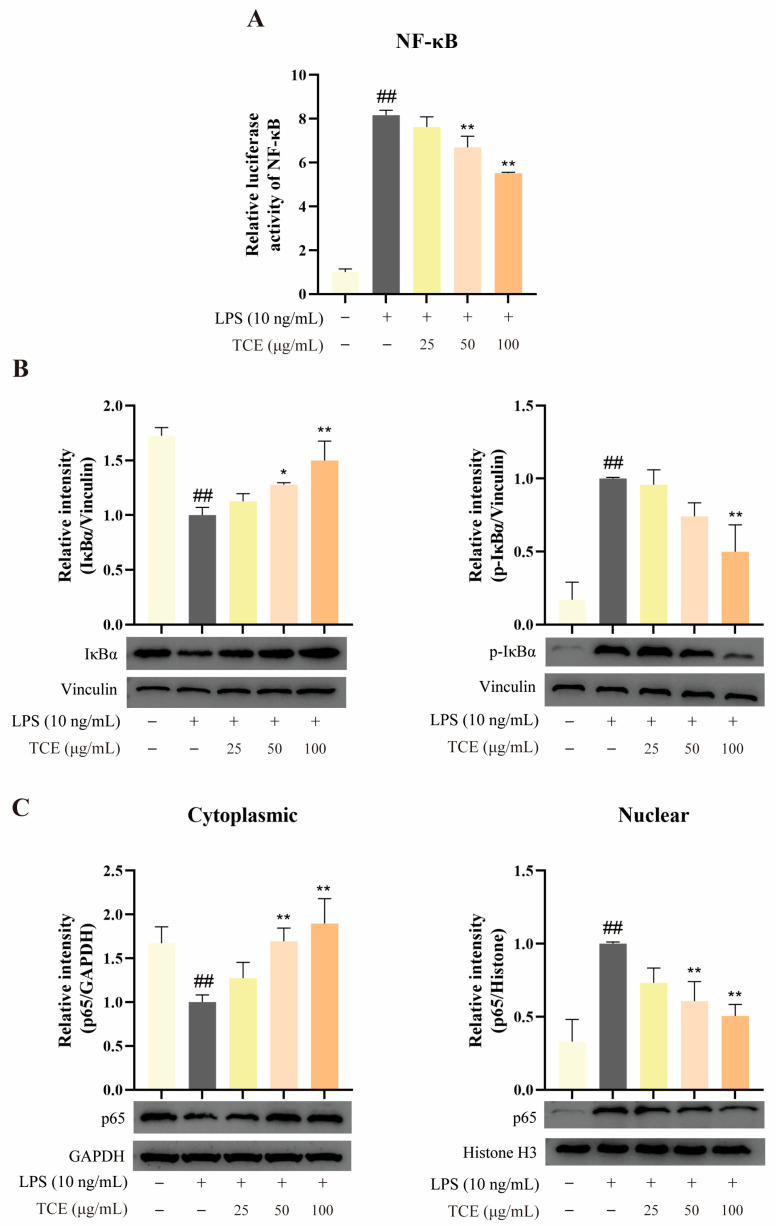
Effect of TCE on NF-κB signaling in LPS-challenged RAW264.7 cells (*n* = 3). (**A**) Effect of TCE on LPS-induced NF-κB activation. (**B**) Effects of TCE on LPS-induced phosphorylation and degradation of IκBα. (**C**) Effect of TCE on the nuclear translocation of p65. ^##^ *p* < 0.01 vs. negative control (vehicle); * *p* < 0.05 and ** *p* < 0.01 vs. LPS alone.

During the immune response, NF-κB functions as a key regulator in the transcriptional induction of inflammation-related genes. To determine whether TCE interferes with the NF-κB pathway, we performed a luciferase reporter assay. The results revealed that TCE exerts a concentration-dependent inhibitory effect on LPS-induced NF-κB activation (Figure 9A). Next, Western blot analysis was carried out to detect the levels of key proteins in NF-κB signaling. As shown in Figure 9B,C, LPS caused a robust decrease in IκBα and an increase in p-IκBα in cytoplasm, accompanied by nuclear p65 rising and cytoplasmic p65 dropping; TCE markedly hindered the phosphorylation and degradation of IκBα. Moreover, it also concentration-dependently prevented the nuclear translocation of p65. These data indicate that TCE exerts a suppressive effect on NF-κB pathway activation following LPS stimulation.

#### 2.4.5. TCE Inhibits AP-1 Activation in LPS-Activated Macrophages

**Figure 10:**
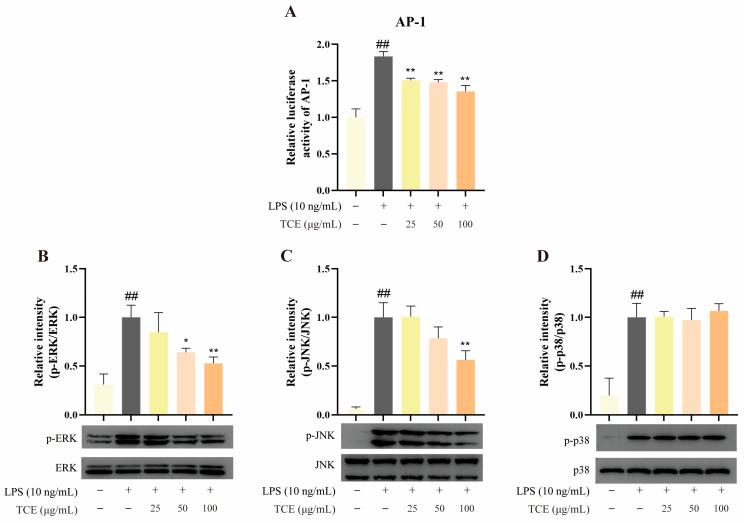
Effect of TCE on AP-1 signaling in LPS-challenged RAW264.7 cells (*n* = 3). (**A**) Effect of TCE on LPS-induced AP-1 activation. (**B**–**D**) Effects of TCE on LPS-induced phosphorylation of ERK (p-ERK), JNK (p-JNK) and p38 (p-p38). ^##^ *p* < 0.01 vs. negative control (vehicle); * *p* < 0.05 and ** *p* < 0.01 vs. LPS alone.

AP-1, another well-known transcription factor, is activated by the phosphorylation of the upstream kinases: ERK, JNK, and p38 [34]. Thus, we next evaluated the effect of TCE on AP-1 signaling. The result of luciferase assay revealed that TCE significantly dampened LPS-induced AP-1 activation (Figure 10A). Furthermore, LPS markedly induced the phosphorylation of ERK, JNK, and p38, while TCE could reduce the levels of p-ERK and p-JNK, but failed to decrease p-p38 (Figure 10B–D). These results demonstrated that TCE exerted its suppressive effect on AP-1 signaling through blocking the phosphorylation events of ERK and JNK.

### 2.5. Anti-Inflammatory Activities of TCE In Vivo

#### 2.5.1. TCE Ameliorates Endotoxemia Mice

**Figure 11:**
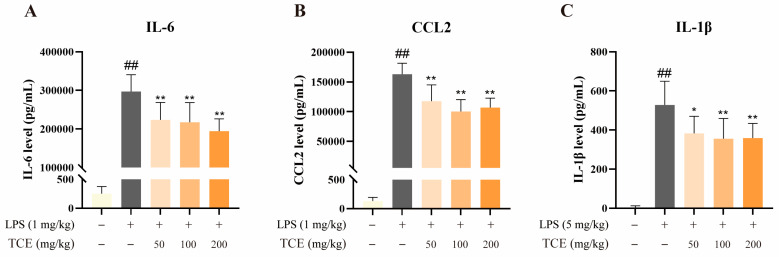
Effects of TCE on the levels of serum pro-inflammatory cytokines in endotoxemia mice (*n* = 8). (**A**) Effect of TCE on serum IL-6 level in endotoxemia mice. (**B**) Effect of TCE on serum CCL2 level in endotoxemia mice. (**C**) Effect of TCE on serum IL-1β level in endotoxemia mice. ^##^ *p* < 0.01 vs. negative control; * *p* < 0.05 and ** *p* < 0.01 vs. LPS alone.

Based on the anti-inflammatory activity of TCE observed in vitro, we further evaluated its efficacy in vivo using a mouse endotoxemia model. As shown in Figure 11A,B, intravenous injection of LPS (1 mg/kg) markedly elevated serum levels of IL-6 and CCL2 2 h after the challenge. In contrast, a single administration of TCE (i.p., 50–200 mg/kg) significantly decreased the levels of these pro-inflammatory cytokines in serum. To detect serum IL-1β in endotoxemia mice, a higher LPS dose (5 mg/kg) was used, and serum was collected 3 h post-injection. As illustrated in Figure 11C, TCE also significantly decreased the serum IL-1β level dose-dependently. These data indicate that TCE has a notable capacity to reduce LPS-elevated pro-inflammatory cytokines in serum, which is consistent with the in vitro findings.

#### 2.5.2. TCE Alleviates LPS-Induced Acute Lung Injury (ALI) in Mice

**Figure 12:**
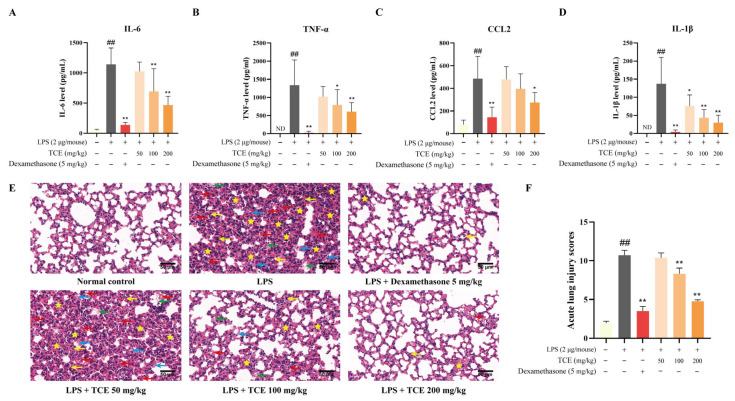
TCE alleviates LPS-induced acute lung injury (ALI) in mice. Effects of TCE on the levels of IL-6 (**A**), TNF-α (**B**), CCL2 (**C**), and IL-1β (**D**) in BALF (*n* = 8). (**E**) Lung injury scores were calculated according to the predetermined criteria. ^##^ *p* < 0.01 vs. negative control group; * *p* < 0.05 and ** *p* < 0.01 vs. model control group. ND, not detected. (**F**) Representative images of H&E staining in lung tissues (200×). Edema is indicated by green arrows, while blue arrows denote alveolar and interstitial inflammation. Red arrows highlight alveolar and interstitial hemorrhage, and yellow arrows mark neutrophil infiltration. The yellow pentagrams point to atelectasis. Scale bars = 50 μm. Dexamethasone served as a positive control.

To investigate the protective effect of TCE on ALI, we established a mouse ALI model via intratracheal delivery of LPS. As shown in Figure 12A–D, the levels of IL-6, tumor necrosis factor alpha (TNF-α), CCL2, and IL-1β in bronchoalveolar lavage fluid (BALF) were robustly increased 6 h after the LPS challenge (2 μg/mouse), while single administration of TCE (50–200 mg/kg, i.p.) resulted in a substantial reduction in these proinflammatory cytokine levels. Histopathological examination further revealed that LPS additionally induced marked pathological alterations in the lung tissues, characterized by thickened alveolar septa, interstitial edema, alveolar hemorrhage, and extensive neutrophil infiltration, along with a marked increase in the lung injury score [35]; meanwhile, TCE exhibited notable improvements in these pathological changes (Figure 12E,F). Together, these observations demonstrate that TCE confers marked protection against LPS-triggered ALI in mice.

## 3. Discussion

*T. chinensis* has been widely used for promoting measles eruption with a long history. Modern phytochemical investigations have revealed that *T. chinensis* is rich in flavonoids, triterpenes, organic acids, and volatile oils [25]. Previous studies have demonstrated that the macromolecules in *T. chinensis*, such as polysaccharides, exhibit significant anti-complement and anti-oxidant activities in vitro [18,27]. According to traditional medicinal practice, *T. chinensis* is commonly administered orally with wine or applied externally for washing, suggesting that its pharmacological activity is primarily attributable to its small-molecule components. Therefore, 85% ethanol reflux was used to extract the small-molecule compounds, as macromolecules are generally insoluble under these conditions. HPLC fingerprint analysis identified three flavonoids in the TCE: quercetin (0.239%), kaempferol (0.033%), and isorhamnetin (0.080%) (Figure 2). Given the well-documented anti-complementary, anti-inflammatory, and anti-oxidative properties of flavonoids (e.g., quercetin and kaempferol) [36,37,38] and phenolic acids (e.g., gallic acid) [39], this study systematically investigated these potential effects of TCE.

Besides the classical pathway, both the lectin and alternative pathways are capable of initiating the complement system [40]. Our results demonstrated that TCE selectively inhibited the classical and lectin pathway-mediated complement activation (Figure 3A,B) but failed to suppress the alternative pathway, even at high concentrations (Figure 3C). Given that the classical and lectin pathways share C4b2a as their C3 convertase, different from the C3 convertase (C3bBb) responsible for the alternative pathway [41], it is reasonable to speculate that the diverse effects of TCE on three complement activation pathways are attributed to the suppression of C4b2a rather than C3bBb.

Oxidative stress is a key initiator of inflammatory responses, and the elimination of excessive ROS is an important strategy for anti-inflammatory therapy [42]. In the cell-free anti-oxidative assay, TCE exhibited robust ferric reducing ability, as well as DPPH radical and superoxide anion scavenging activities (Figure 4A–C), showing a comprehensive free radical scavenging ability. In contrast, its scavenging activity on hydroxyl radical was limited (Figure 4D), which may be attributed to the weak metal-chelating capacity of its major active constituents (e.g., flavonoids and phenolic acids), thus failing to effectively inhibit the Fenton reaction. In the cellular anti-oxidative assay, at non-toxic concentrations (Figure 5A), TCE substantially inhibited LPS-induced intracellular ROS accumulation (Figure 5B). The generation of ROS is primarily attributable to two distinct sources: the electron transport chain situated within mitochondria, and a family of membrane-bound enzymatic complexes collectively referred to as NADPH oxidases (NOXs) [43]. Our results demonstrated that TCE suppressed NADPH oxidase activity but had no obvious effect on mitochondrial ROS production (Figure 5C,D). These findings indicate that TCE exerts intracellular anti-oxidative effects that may involve two aspects: (1) inhibiting NADPH oxidase activity to decrease endogenous superoxide anion production; (2) directly scavenging superoxide anions to lower intracellular ROS levels.

Inflammatory response is driven by the release and subsequent action of proinflammatory mediators. As a key inflammatory mediator, excessive NO produced by iNOS participates in various inflammatory diseases [44]. In macrophages, LPS stimulation induces robust iNOS expression, which is responsible for the production of NO [45]. Enhanced activity and upregulated expression of iNOS can both promote NO production. In this study, TCE, at the non-toxic concentrations, strongly reduced LPS-induced NO production in both RAW264.7 cells and primary MPMs (Figure 6B,C). Regarding iNOS, TCE markedly suppressed its transcription and translation but failed to affect its activity (Figure 7A–C).

Apart from NO, LPS-activated macrophages also produce other proinflammatory cytokines, including IL-6, CCL2, and IL-1β, which are important mediators of inflammatory cascade reactions. IL-6 serves as a major acute-phase mediator, enhancing the intensity of acute inflammation and thereby leading to a febrile response [46,47]. Acting as a chemotactic factor, CCL2 organizes the accumulation of leukocytes at sites of infection or inflammation, a process that perpetuates the local inflammatory state [48]. IL-1β is also a potent pro-inflammatory cytokine that results in tissue destruction [49] and leads to fever and endotoxin shock [50]. It was found that TCE potently reduced the supernatant levels of IL-6 and CCL2 in LPS-activated RAW264.7 cells and MPMs in a concentration-dependent manner (Figure 8A–D). As for the intracellular level of pro-IL-1β, TCE also exhibited a potent inhibitory effect (Figure 8E). At the transcriptional level, TCE markedly downregulated their mRNA levels (Figure 8F–H), indicating that it inhibits the production of these pro-inflammatory cytokines by regulating their gene transcription.

The expression of inflammatory genes is primarily regulated by two major transcription factors: NF-κB and AP-1. In response to LPS stimulation, IκBα undergoes phosphorylation and degradation, which results in the rapid and transient translocation of NF-κB (p65/p50) to the nucleus, where it binds to a specific DNA element and initiates the transcription of inflammatory genes [51,52]. It was demonstrated that TCE inhibited IκBα phosphorylation and prevented the nuclear translocation of p65, thus suppressing NF-κB signaling (Figure 9A–C). In addition to NF-κB, AP-1 is simultaneously activated under LPS exposure. This process depends on downstream intracellular signaling events that lead to the phosphorylation of several MAPK family members, namely, JNK, ERK, and p38. This leads to the activation of AP-1, thus promoting the expression of pro-inflammatory mediators [34]. Our data suggested that TCE could potently suppress LPS-induced AP-1 activation, which was attributed to the inhibition of the phosphorylation of ERK and JNK, but failed to affect p-p38 (Figure 10A–D). Given that the pro-inflammatory mediators are well-established downstream target genes of both NF-κB and AP-1 [53], the suppression of these two signaling by TCE directly accounts for the reduced mRNA and protein levels of these pro-inflammatory mediators (e.g., iNOS, IL-6, CCL2, and IL-1β). These effects might be related to the fact that some of its constituents (e.g., quercetin, kaempferol, gallic acid, ellagic acid, syringic acid) possess similar activities [38,39,54,55,56].

## 4. Materials and Methods

### 4.1. Reagents

DMEM (Cat^#^12100046, Lot^#^2503650) was obtained from Gibco BRL (Grand Island, NY, USA). Fetal bovine serum (FBS) (Cat^#^11011-8611, Lot^#^25010703) was produced by Zhejiang Tianhang Biotechnology Co. (Hangzhou, Zhejiang, China). LPS (Cat^#^L4130, Lot^#^0000130726), Apocynin (Cat^#^A10809, Lot^#^STBC0387), Zymosan A (Cat^#^Z4250, Lot^#^102710637), lucigenin (Cat^#^M8010, Lot^#^MKBB2539), and L-NAME (Cat^#^N55751, Lot^#^102856416) were purchased from Sigma-Aldrich (St. Louis, MO, USA). Mito-TEMPO (Cat^#^HY-112879, Lot^#^41776) was from MedChemExpress (Monmouth Junction, NJ, USA). Celecoxib (Cat^#^C9180, Lot^#^119F021) and NADPH (Cat^#^N8100, Lot^#^24230825001) were obtained from Solarbio Co. (Beijing, China). Cell Counting Kit-8 (Cat^#^GK10001, Lot^#^114) was from Glpbio Technology (Montclair, CA, USA). L-012 (Cat^#^12004891, Lot^#^CTN7966) was from Wako Chemicals (Tokyo, Japan). Dexamethasone (Cat^#^S17003, Lot^#^YO7D6C7163) and Mannan (Cat^#^S51698, Lot^#^S15F10T78556) were obtained from Shanghai Yuanye Bio-Technology Co. (Shanghai, China). Normal Human Serum Complement (Cat^#^A112, Lot^#^230610) was from Quidel Corporation (San Diego, CA, USA). C5b-9 (aE11) (Cat^#^sc-58935, Lot^#^D2125) was from Santa Cruz Biotechnology (Dallas, TX, USA). Human IgM (Cat^#^bs-0345P, Lot^#^BE03219261) was from Bioss Inc. (Woburn, MA, USA). Nuclear and cytoplasmic protein extraction kit (Cat^#^P0028, Lot^#^022724240531), luciferase assay system (Cat^#^RG055, Lot^#^A578260311), and monoclonal antibodies against vinculin (Cat^#^AG3539, Lot^#^Z954250324) were acquired from Beyotime Biotechnology (Shanghai, China). TRIzol reagent (Cat^#^P0028, Lot^#^C706K133212) and MitoSOXTM Red mitochondrial superoxide indicator (Cat^#^M36008, Lot^#^2530145) were from Invitrogen Co. (Carlsbad, CA, USA). EasyScript All-in-One First-Strand cDNA Synthesis SuperMix for qPCR (Cat^#^AE341-02, Lot^#^S3250622) was from TransGen Biotech (Beijing, China). SYBR^®^ FAST qPCR Kit (Cat^#^KK4602, Lot^#^0000126131) was from Kapa Biosystems Pty, Ltd. (Boston, MA, USA). Mouse IL-1β (Cat^#^432604, Lot^#^B352628), IL-6 (Cat^#^431301, Lot^#^B371164), and CCL2 (Cat#432701, Lot#B356861) ELISA kits were from BioLegend Co. (San Diego, CA, USA). Mammalian protein extraction kit (Cat^#^CW0889M, Lot^#^01364/34020) was obtained from Cowin Bio. (Taizhou, Jiangsu, China). Antibodies against iNOS (Cat^#^13120, Lot^#^5), NF-κB p65 (Cat^#^4764S, Lot^#^8), p-IκBα (Cat^#^2859S, Lot^#^18), p-ERK (Cat^#^4370S, Lot^#^30), JNK (Cat^#^9258S, Lot^#^11), p-JNK (Cat^#^9251S, Lot^#^27), p38 (Cat^#^9212S, Lot^#^26), p-p38 (Cat^#^9215S, Lot^#^7) and Histone H3 (Cat^#^9715S, Lot^#^24) were acquired from Cell Signaling Technology (Danvers, CO, USA). Antibodies against ERK (Cat^#^A10613, Lot^#^9410613001), GAPDH (Cat^#^AC033, Lot^#^9100033001), and horseradish peroxidase (HRP)-conjugated anti-mouse (Cat^#^AS003, Lot^#^9300033001); anti-rabbit IgG (Cat^#^AS014, Lot^#^9300014001); ColorMixed Protein Marker 180 (10–180 kDa) (Cat^#^RM19001, Lot^#^9623141422); and mouse TNF-α ELISA kit (Cat^#^RK00027, Lot^#^9680058060126) were bought from ABclonal Biotech Co. (Wuhan, Hubei, China). Compstatin (Cat^#^C860471, Lot^#^C1512264) was obtained from Macklin Biochemical (Shanghai, China). All other reagents were of analytical grade.

### 4.2. Cell Isolation and Culture

The murine macrophage cell line (RAW264.7) was obtained from American Type Culture Collection (ATCC, Rockville, MD, USA). Mouse peritoneal macrophages (MPMs) were isolated from male ICR mice 3 days after intraperitoneal injection of 1 mL of 3.8% thioglycolate medium in accordance with the procedure described previously [57]. Both kinds of macrophages were cultured in DMEM containing 10% heat-inactivated FBS and 1% penicillin–streptomycin solution in a humidified 5% CO_2_ atmosphere at 37 °C.

### 4.3. Animals and Ethical Statements

The male ICR mice (SPF grade, 18–22 g) were purchased from Beijing HFK Bio-Technology Co., Ltd. (Beijing, China; license number: SCXK (Beijing) 2023-0008). The mice were housed in a pathogen-free barrier facility with a 12 h light on/off cycle and standard laboratory temperature and humidity conditions. The experimental procedures were conducted in accordance with the ARRIVE guidelines and were approved by the Institutional Care and Use Committee of the Institute of Medicinal Plant Development (IMPLAD) of the Chinese Academy of Medical Sciences (No. SLXD-20250102013). Anesthetic drugs and all other necessary measures were used to alleviate animal suffering during the experimental procedures.

### 4.4. Preparation of TCE

The delicate branches and leaves of *Tamarix chinensis* Lour. were collected from the Institute of Medicinal Plant Development, Beijing, China (40°2′4.73″ N, 116°16′21.30″ E), in September, 2022, and identified by Prof. Yulin Lin (Institute of Medicinal Plant Development, Chinese Academy of Medical Sciences and Peking Union Medical College, Beijing, China). The voucher specimen (No. BJ-2022-LC-365) was deposited in the Institute of Medicinal Plant Development, Chinese Academy of Medical Sciences & Peking Union Medical College. The delicate branches and leaves were air-dried in the shade to remove all of the moisture. Dry samples were dipped in 85% ethanol solution (1:20, *w*/*v*) overnight and then extracted twice via refluxing for 2 h each time. The obtained extract (TCE) was filtered and concentrated under reduced pressure by using a rotary evaporator (N-1100, EYELA, Tokyo, Japan). The residue was dried to a constant weight with a yield of 17.18% (*w*/*w*). The final sample was stored at −20 °C for further use.

### 4.5. HPLC Analysis of TCE

The HPLC assay was applied as previously described with slight modifications [58]. An LC-15C HPLC system (Shimadzu, Kyoto, Japan) equipped with a UV spectrophotometer detector (Shimadzu, Japan) and packed with a reversed-phase SyncronisTM C18 column (4.6 mm × 250 mm, 5 μm; Thermo Fisher Scientific, Waltham, MA, USA) was used for HPLC analysis. The mobile phases were methanol (A) and 0.1% phosphoric acid aqueous solution (B). The samples were eluted with a gradient as follows: 22% A (0–10 min); 22–32% A (10–30 min); 32% A (30–45 min); 32–52% A (45–65 min); 52–72% A (65–74 min); 72–85% A (74–85 min); 85% A (85–90 min). The flow rate was 1.0 mL/min, and the column temperature was at room temperature. The UV spectrophotometer detector was set at 360 nm. The components in TCE were identified by comparing the retention time with that of standard compounds and then quantified using the external standard method.

### 4.6. Cell Viability Assay

Cell viability was determined using a Cell Counting Kit-8 (CCK-8) assay according to the manufacturer’s instructions. In brief, RAW264.7 cells (4 × 10^5^ cells per well) or MPMs (1 × 10^6^ cells per well) were cultured in 96-well plates. Cells were incubated with different concentrations of TCE (25, 50, 100, 200, 400, 600 and 800 μg/mL) for 22 h and followed by adding CCK-8 solution for a further 2 h. The optical density at 450 nm was measured with a microplate reader (Thermo Fisher Scientific, USA).

### 4.7. Measurement of Anti-Complement Activity

The anti-complement activity of TCE was evaluated using an ELISA-based system following a previously established method [29]. In brief, to initiate the classical, alternative, and lectin pathways, high-binding ELISA plates were coated with 1 μg/mL human immunoglobulin M (IgM), 1 mg/mL zymosan, or 100 μg/mL mannan, respectively, overnight at 4 °C, and then blocked with 1% BSA for subsequent use. Normal human serum (NHS) was pretreated with different concentrations of TCE for 15 min at room temperature, then 25 μL of each mixture was transferred to the corresponding coated ELISA plates and incubated for 30 min at 37 °C to initiate complement activation. After washing with PBST 4 times to terminate the reaction, C5b-9 deposition was detected using an anti-human C5b-9 antibody for 1 h at room temperature, followed by HRP-conjugated Donkey anti-Mouse IgG antibody for 30 min at room temperature. Finally, TMB substrate was added, and the optical densities were measured at 450 nm to quantify C5b-9 levels. Compstatin was used as a positive control.

### 4.8. Measurement of Anti-Oxidative Capacity in Cell-Free Systems

The ferric reducing ability, DPPH radical scavenging activity, and superoxide anion scavenging activity of TCE were assessed following an established protocol [59]. Ferric reducing ability was expressed as Trolox (μM) equivalent anti-oxidant capacity, where Trolox is a hydrophilic analog of vitamin E. The hydroxyl radical scavenging activity of TCE was determined using a previously established method [60].

### 4.9. Measurement of Anti-Oxidative Capacity in Cell System

#### 4.9.1. Measurement of Intracellular ROS

The intracellular ROS levels were measured with the chemiluminescent probe L-012, following a previously established method [61]. In brief, RAW264.7 macrophages were plated at a density of 4 × 10^5^ cells per well in a white 96-well cell culture plate and were pretreated with different concentrations of TCE (25, 50, and 100 μg/mL) or Apocynin (200 μM) for 1 h and then stimulated and incubated with LPS (10 ng/mL) for 12 h. Then, 100 μL of the L-012 probe (100 μM) was added and incubated at 37 °C for 30 min. The chemiluminescence was detected by using a microplate counter (MicroBeta2, PerkinElmer, Waltham, MA, USA). Apocynin was used as a positive control.

#### 4.9.2. Measurement of Intracellular NADPH Oxidase Activity

The intracellular NADPH oxidase activity was detected following a previously established method [61]. RAW264.7 macrophages were seeded in 6-well plates (density, 8 × 10^6^ cells per well). After pretreatment with different concentrations of TCE (25, 50, and 100 μg/mL) or Apocynin (200 μM) for 1 h, the cells were stimulated with LPS (10 ng/mL) for 6 h, then they were washed twice with PBS and collected. Then, cells were scraped from the plate and were sonicated on ice in a buffer containing 1 mM EDTA, protease inhibitor cocktail, and 50 mM KH_2_PO_4_ (pH 7.0). Following centrifugation of the cell lysate (13,000× *g*, 4 °C, 5 min), supernatant was collected for protein concentration determination using a BCA assay. Subsequently, 20 μL of the supernatant was transferred to a white 96-well plate and incubated with 80 μL lucigenin at 25 °C in the dark for 5 min. The chemiluminescence signal was measured immediately by using a microplate counter (MicroBeta2, PerkinElmer, USA) following the addition of 100 µL of NADPH (1 mM in 50 mM KH_2_PO_4_ buffer, pH 7.0). Apocynin was used as a positive control.

#### 4.9.3. Measurement of Mitochondrial ROS (mtROS)

The mtROS levels were measured following a previously established method [62]. RAW264.7 macrophages were plated in a black 96-well plate at a density of 2 × 10^4^ cells per well. Cells were pretreated with different concentrations of TCE (25, 50, and 100 μg/mL) or with Mito-TEMPO (60 μM) for 1 h, followed by stimulation with LPS (10 ng/mL) for 24 h. After washing cells twice with D-Hank’s buffer, the cells were incubated with 100 μL of MitoSOX Red (5 μM) at 37 °C for 30 min. After washing the cells two times with D-Hank’s buffer, the fluorescence intensity was measured at λex 510 nm and λem 590 nm using a fluorescence microplate reader (Fluoroskan Ascent FL, Thermo Fisher Scientific, Waltham, MA, USA). Mito-TEMPO was used as a positive control.

### 4.10. Measurement of NO Production

The level of NO production was monitored by measuring the nitrite level in the culture medium using the Griess reaction, as previously reported [63]. RAW264.7 cells (4 × 10^5^ cells per well) or MPMs (1 × 10^6^ cells per well) were seeded in 96-well plates. Cells were pretreated with TCE (25, 50, and 100 μg/mL) or L-NAME (100 μM) for 1 h and then stimulated by LPS (10 ng/mL) for another 24 h. The nitrite production was measured by mixing 100 μL of supernatant and 100 μL of Griess reagent. The optical densities were measured at 540 nm. The nitrite concentration was calculated with reference to a standard curve.

### 4.11. Measurement of iNOS Activity

Intracellular iNOS activity was measured as previously described with slight modifications [61]. RAW264.7 cells were exposed to LPS (10 ng/mL) for 12 h to induce intracellular iNOS formation. After being washed three times by D-Hank’s solution, the activated cells were subsequently seeded in 48-well plates and treated with TCE at different concentrations for another 12 h. The iNOS activity was assayed by measuring the supernatant NO production.

### 4.12. Measurement of Pro-Inflammatory Cytokines

RAW264.7 cells (4 × 10^5^ cells per well in 96-well plates) or MPMs (1 × 10^6^ cells per well in 96-well plates) were pretreated with designated TCE or celecoxib (50 μM) for 1 h and followed by LPS (10 ng/mL) stimulation for 24 h. IL-1β in the lysates and MCP-1 and IL-6 in the supernatants were determined using ELISA kits according to the manufacturer’s instructions. The concentrations of pro-inflammatory mediators were calculated from their respective standard curves. Celecoxib was used as the positive control.

### 4.13. RNA Extraction and Quantitative Real-Time PCR (RT-qPCR)

RAW264.7 cells were seeded at a density of 1 × 10^7^ cells per well in 6-well plates for 24 h in a humidified incubator with 5% CO_2_ at 37 °C. Cells were pretreated with TCE at different concentrations for 1 h and then stimulated by LPS (10 ng/mL) for 4 h. Total mRNA was isolated from RAW264.7 cells by Trizol reagent. cDNA was synthesized from 1 μg of total RNA using TransScript All-in-One First-Strand cDNA Synthesis SuperMix. RT-*q*PCR analyses were performed using SYBR^®^ FAST *q*PCR Kit according to the manufacturer’s instructions with the primer sequences (Table 1) [64]. The levels of iNOS, IL-1β, IL-6 and MCP-1 mRNA were normalized to GAPDH.

### 4.14. Plasmids Transfection and Luciferase Reporter Assay

RAW264.7 cells were stably transfected with plasmid including pNFκB-TA-luc or pAP1-TA-luc by Entranster™-H4000 (Engreen Biotechnology, Beijing, China) according to the manufacturer’s instructions. The obtained cells were seeded at a density of 2.5 × 10^6^ per well in 24-well plates with different concentrations of TCE for 1 h and followed by LPS (10 ng/mL) stimulation for 4 h. The cells were then lysed for the luciferase activity assay according to the manufacturer’s protocol by using a microplate counter (MicroBeta2, PerkinElmer, Waltham, MA, USA).

### 4.15. Western Blot

RAW264.7 cells were seeded in 6-well plates (1 × 10^7^ cells per well). The cells were pretreated with TCE at various concentrations for 1 h and stimulated by LPS (10 ng/mL) for different times. Total proteins were extracted using the mammalian protein extraction kit. Nuclear and cytoplasmic proteins of cells were extracted using the nuclear and cytoplasmic protein extraction kit. Equivalent proteins (20 μg per lane) were separated by SDS-PAGE and semi-dry transferred to PVDF membranes. Subsequently, the membranes were blocked with 5% non-fat milk powder in TBST at room temperature for 2 h and then incubated with each primary antibody overnight at 4 °C. After washing three times with TBST, the membranes were incubated with HRP-conjugated anti-rabbit or mouse IgG secondary antibodies for 1 h at room temperature. After repeating the above washing process, the protein bands were detected using an ECL Western blotting kit and photographed by a ChemiDoc XRS+ imaging system (Bio-Rad Technology, Hercules, CA, USA). Quantification of Western blots was performed using ImageJ software (version 1.53e; Stuttgart, Baden-Württemberg, Germany).

### 4.16. Endotoxemia Mice Model

The in vivo anti-inflammatory effect of TCE was explored as previously described [65]. Male ICR mice were randomly assigned to 5 groups (*n* = 8): negative control group (vehicle), model group (1 mg/kg LPS), and TCE groups (50 mg/kg TCE, 100 mg/kg TCE, or 200 mg/kg TCE + 1 mg/kg LPS). TCE dissolved in saline containing DMSO (5%, *v*/*v*) and Tween-80 (2%, *v*/*v*) was administered intraperitoneally 30 min before intravenous injection of LPS, while negative control and model groups received an equal volume of vehicle. Two hours later, blood was collected and placed in 4 °C overnight. Serum pro-inflammatory cytokines were measured by ELISA.

### 4.17. LPS-Induced Acute Lung Injury (ALI) Mice Model

An LPS-induced ALI mice model was performed as previously established protocol [66]. Briefly, male ICR mice were randomly divided into 6 groups (*n* = 11): negative control group (vehicle), model group (2 μg/mouse LPS), TCE groups (50 mg/kg TCE, or 100 mg/kg TCE, or 200 mg/kg TCE + 2 μg/mouse LPS), and positive control group (5 mg/kg dexamethasone + 2 μg/mouse LPS). TCE dissolved in saline containing DMSO (5%, *v*/*v*) and Tween-80 (2%, *v*/*v*) was administrated intraperitoneally 30 min before the LPS challenge. Subsequently, the mice were anesthetized with 2% isoflurane, and ALI mice were then induced via an intratracheal instillation of LPS (2 μg/20 μL per mouse) using a Mouse MicroSprayer Aerosolizer (BioJane, Shanghai, China). Control group mice received an equivalent volume of normal saline instead. Then, 6 h after the LPS challenge, all mice were anesthetized again and humanely euthanized for sample collection.

Three mice were randomly chosen from each group and sacrificed for pathological examination of the lung injury. Their intact lung tissues were fixed in 4% paraformaldehyde, embedded in paraffin, sectioned at 3 μm, and stained with hematoxylin and eosin (H&E). Histopathological evaluation was observed using a digital slide scanner (3Dhistech, Pannoramic MIDI, Hungary). To quantify the severity of the lung injury, six high-power fields (200× magnification) per mouse were selected at random for analysis. All fields were blindly analyzed and scored based on Smith’s scoring method with slight modifications [35]. The scoring system included the following parameters: (I) pulmonary edema, (II) alveolar and interstitial inflammation, (III) alveolar and interstitial hemorrhage, and (IV) atelectasis and formation of the hyaline membrane. Each parameter was assigned a score from 0 to 4, corresponding to the percentage of the field affected: 0 (no injury), 1 (mild, injury in 25% of the field), 2 (moderate, injury in 50%), 3 (severe, injury in 75%), and 4 (profound, injury in entire field). Subsequently, the total lung injury score was derived by summing the scores for all individual parameters.

To determine the inflammatory cytokine levels in ALI mice, bronchoalveolar lavage fluid (BALF) was collected from the remaining mice via intratracheal intubation and lung lavage (three times with 1 mL of ice-cold PBS) and then analyzed using commercial ELISA kits.

### 4.18. Statistical Analysis

Data were analyzed using GraphPad Prism Version 8.0 (GraphPad Software, Inc., La Jolla, CA, USA), and results were presented as mean ± SD of at least three independent experiments. A Student’s *t*-test was used for comparing two groups, and one-way ANOVA followed by the Tukey’s post-test was used for comparing more than two groups. *p* < 0.05 was considered statistically significant.

## 5. Conclusions

**Figure 13:**
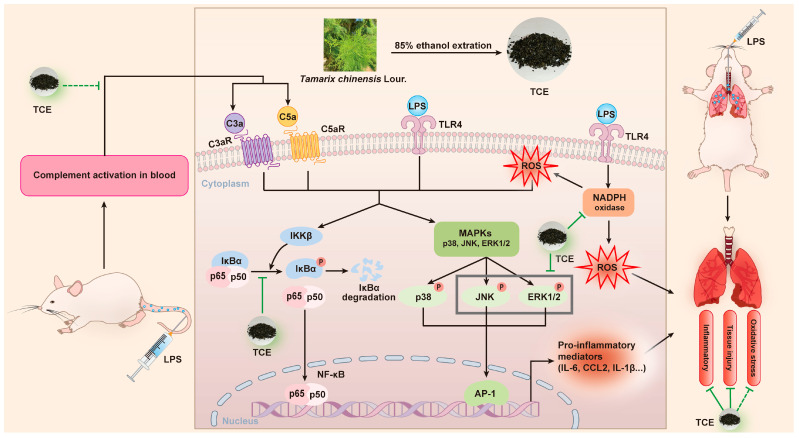
Schematic diagram of the molecular mechanisms of TCE. Solid green lines indicate experimentally validated inhibitory effects. Dashed green lines indicate possible inhibitory effects that remain to be verified.

Taken together, the present study investigated the anti-complement, anti-oxidant, and anti-inflammatory properties of *T. chinensis*. TCE selectively inhibited the classical and lectin pathways, exerted strong anti-oxidative effects in both cell systems and cell-free systems. In LPS-activated macrophages, TCE decreased the expression of pro-inflammatory mediators by suppressing NF-κB and AP-1 pathways. In vivo, it not only reduced serum pro-inflammatory cytokines in endotoxemia mice but also alleviated LPS-induced ALI of mice (Figure 13). Our findings provide a pharmacological basis for the traditional use of *T. chinensis*.

## 6. Limitations and Future Perspectives

Although the present study, guided by traditional applications, has demonstrated the anti-inflammatory, anti-oxidant, and anti-complement activities of TCE, several limitations should be acknowledged. First, regarding the constituents’ basis, only three flavonoids in the TCE were identified. Future work should therefore systematically investigate the full spectrum of chemical constituents, including active metabolites, and establish unambiguous constituent–activity relationships. Second, regarding translational and clinical applicability, *T. chinensis* exhibits both anti-complement and anti-inflammatory properties. Beyond its traditional use in measles, other complement-mediated inflammatory diseases (e.g., glomerulonephritis) may represent potential expanded application of *T. chinensis*. It is a valuable issue to investigate how to utilize its pharmacological actions to guide its more effective use in Chinese medicine formulations. Finally, although preliminary cytotoxicity tests were conducted in the present efficacy study, further comprehensive toxicological evaluation is also warranted to ensure the clinical safety of *T. chinensis*.

## Figures and Tables

**Table 1:** Sequences of primers used in the RT-qPCR analysis.

Gene		Primer Sequences
*iNOS*	F	5′-CTC AGC CCA ACA ATA CAA G-3′
	R	5′-CTA CAG TTC CGA GCG TCA-3′
*IL-1β*	F	5′-GCA ACT GTT CCT GAA CTC AAC T-3′
	R	5′-ACT TTT TGG GGT CCG TCA ACT-3′
*IL-6*	F	5′-CTG CAA GAG ACT TCC ATC CAG-3′
	R	5′-AGT GGT ATA GAC AGG TCT GTT GG-3′
*MCP-1*	F	5′-AGA TGC AGT TAA CGC CCC AC-3′
	R	5′-AGA CCT TAG GGC AGA TGC AG-3′
*GAPDH*	F	5′-GGT TGT CTC CTG CGA CTT CA-3′
	R	5′-TGG TCC AGG GTT TCT TAC TCC-3′

F, forward primer; R, reverse primer; *iNOS*, inducible nitric oxide synthase; *IL-1β*, interleukin-1 beta; *IL-6*, interleukin-6; *MCP-1*, monocyte chemoattractant protein-1; *GAPDH*, glyceraldehyde-3-phosphate dehydrogenase.

## Data Availability

The original contributions presented in this study are included in the article/Supplementary Materials. Further inquiries can be directed to the corresponding authors.

## Supplementary Materials

The following supporting information can be downloaded at: https://www.mdpi.com/article/10.3390/plants15142199/s1.

## Abbreviations

The following abbreviations are used in this manuscript:

ALI	Acute lung injury
AP-1	Activator protein-1
BALF	Bronchoalveolar lavage fluid
CCK8	Cell Counting Kit-8
CCL2	C-C motif chemokine ligand 2
DMSO	Dimethyl Sulfoxide
DPPH	2,2-Diphenyl-1-picrylhydrazyl
ELISA	Enzyme-Linked Immunosorbent Assay
ERK	Extracellular signal-regulated kinase
HPLC	High-Performance Liquid Chromatography
IC50	Half-maximal inhibitory concentration
IgM	Immunoglobulin M
IKKβ	Inhibitor of kappa B kinase bate
IL-1β	Interleukin-1 beta
IL-6	Interleukin-6
iNOS	Inducible nitric oxide synthase
IκBα	Inhibitor of κB alpha
JNK	c-jun N-terminal kinase
LPS	Lipopolysaccharide
MAPKs	Mitogen-activated protein kinases
MPM	Mouse peritoneal macrophage
mtROS	Mitochondrial Reactive Oxygen Species
MyD88	Myeloid differentiation primary response 88
NADPH	Nicotinamide adenine dinucleotide phosphate
NF-κB	Nuclear Factor Kappa-B
NHS	Normal human serum
NO	Nitric oxide
NOX	NADPH oxidase
P38	p38 mitogen-activated protein kinase
PAMP	Pathogen-associated molecular pattern
ROS	Reactive oxygen species
TCE	Ethanol extract of *T. chinensis*
TLR4	Toll-like receptor 4
TNF-α	Tumor necrosis factor alpha
