# Supplementary material for: Anti-Complement, Anti-Oxidative, and Anti-Inflammatory Activities of the Ethanol Extract of Tamarix chinensis Lour
Source: Plants (Basel). 2026 Jul 18;15(14):2199. doi: 10.3390/plants15142199 (PMC13415313; doi:10.3390/plants15142199)

Figure. 7C iNOS

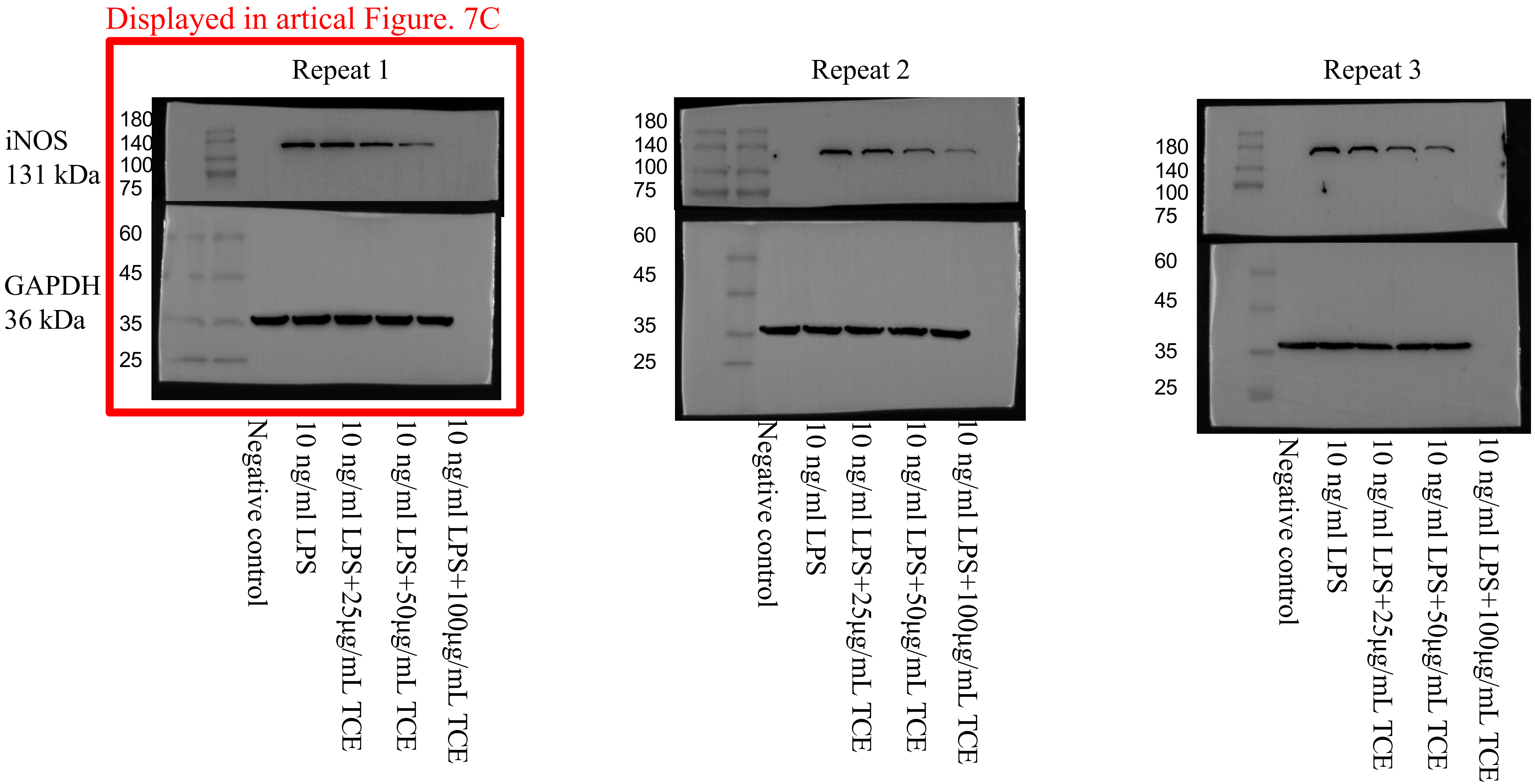

Figure. 9B IκBα

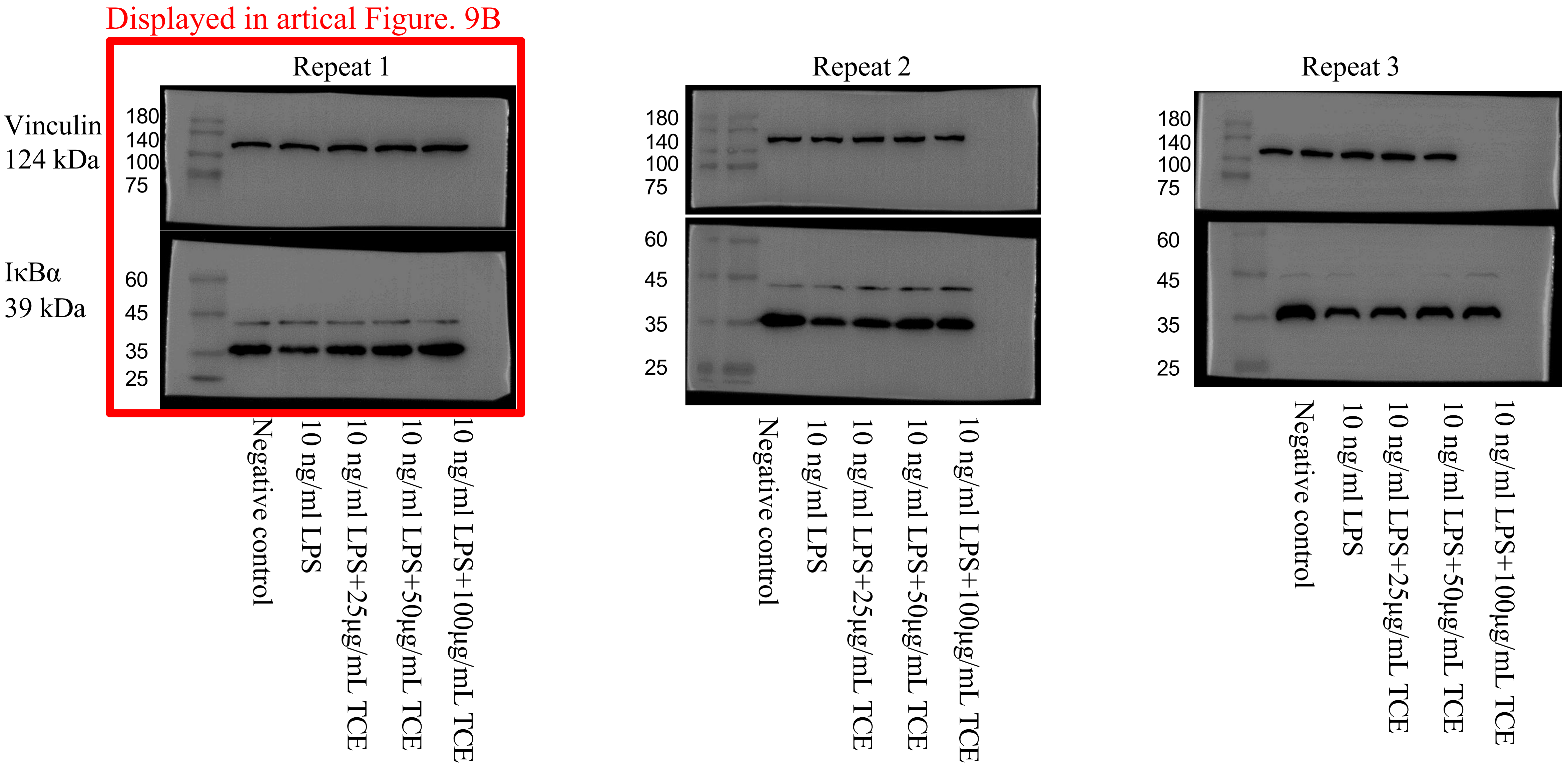

Figure. 9B p-IκBα

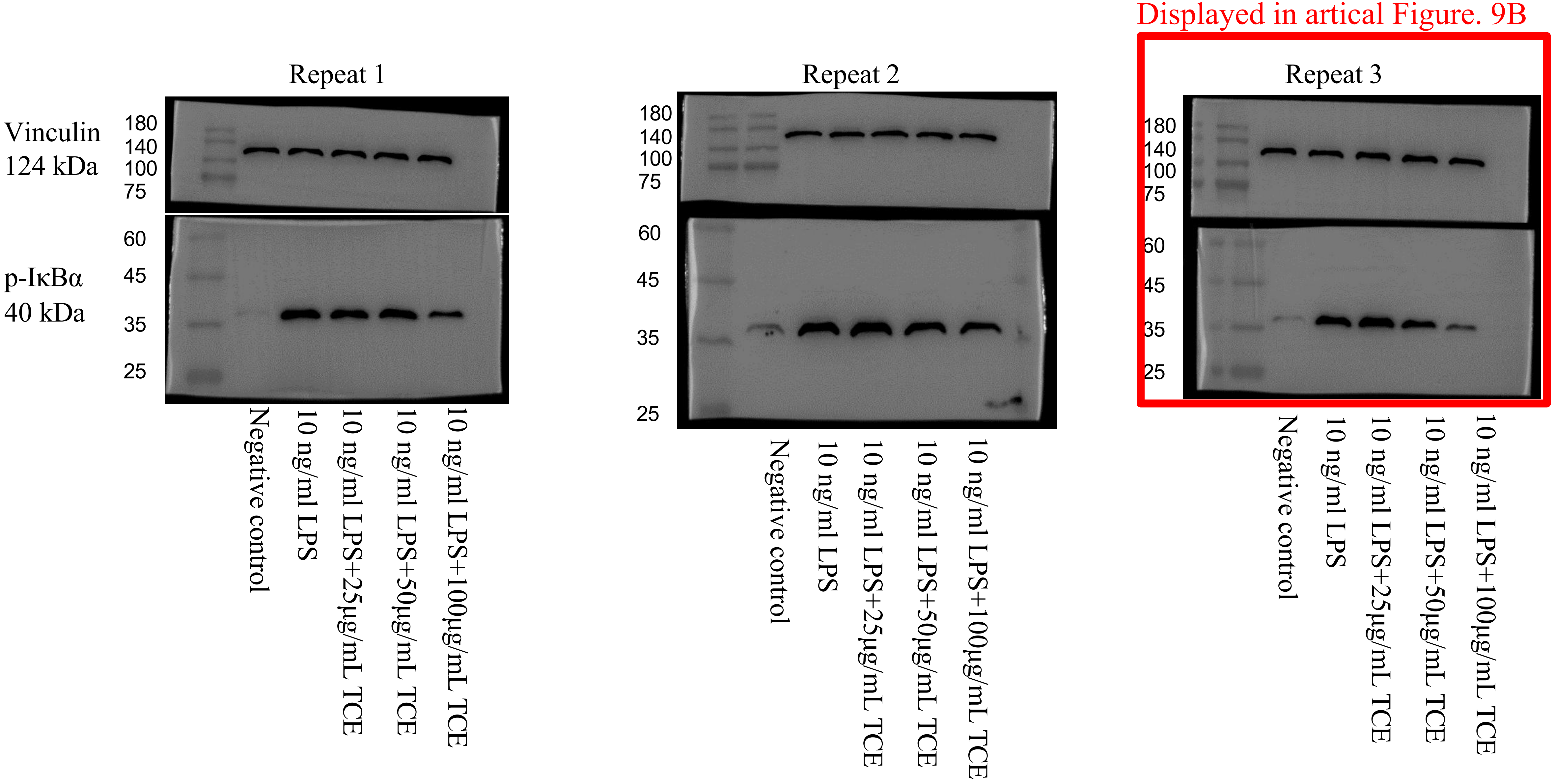

Figure. 9C Cytoplasmic p65

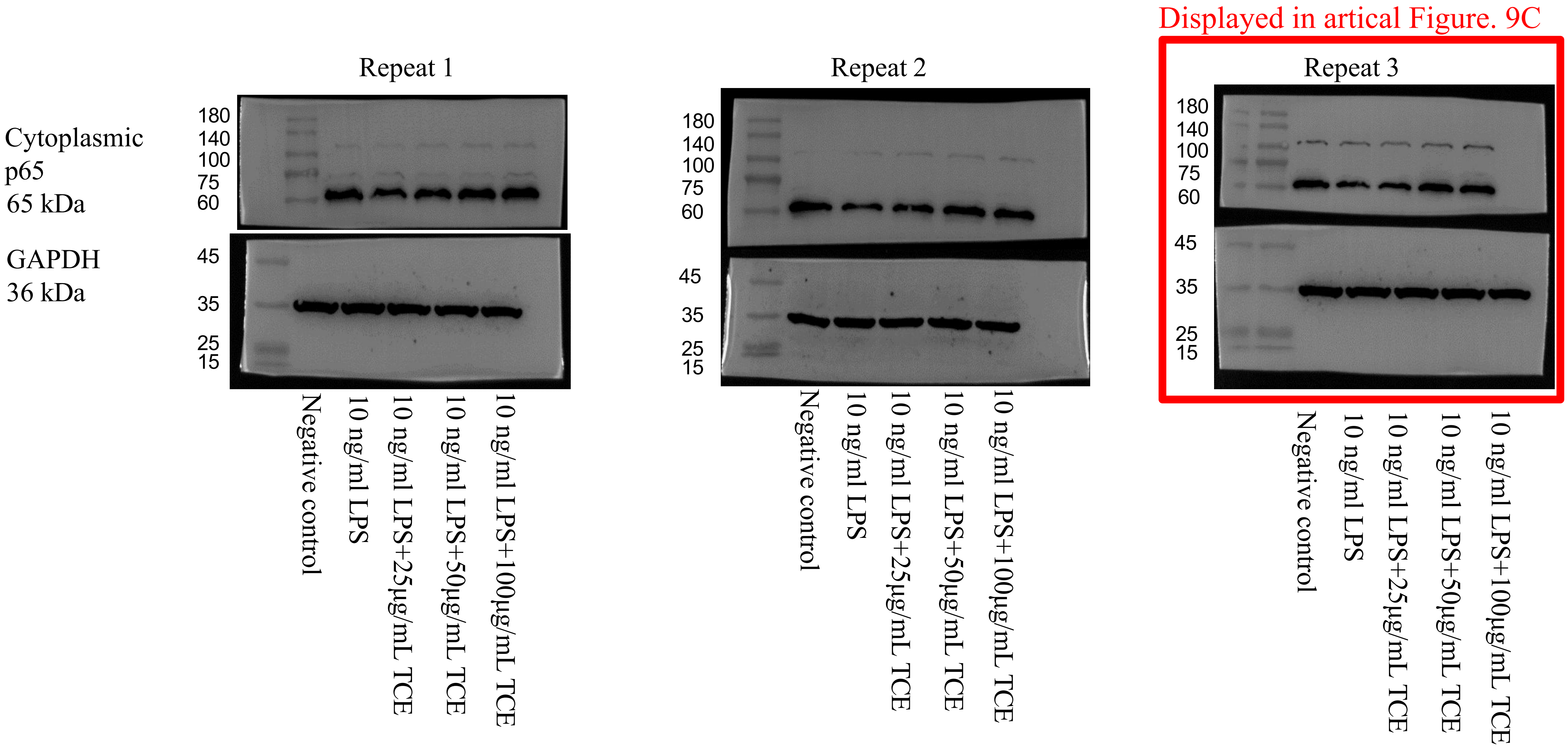

Figure. 9C Nuclear p65

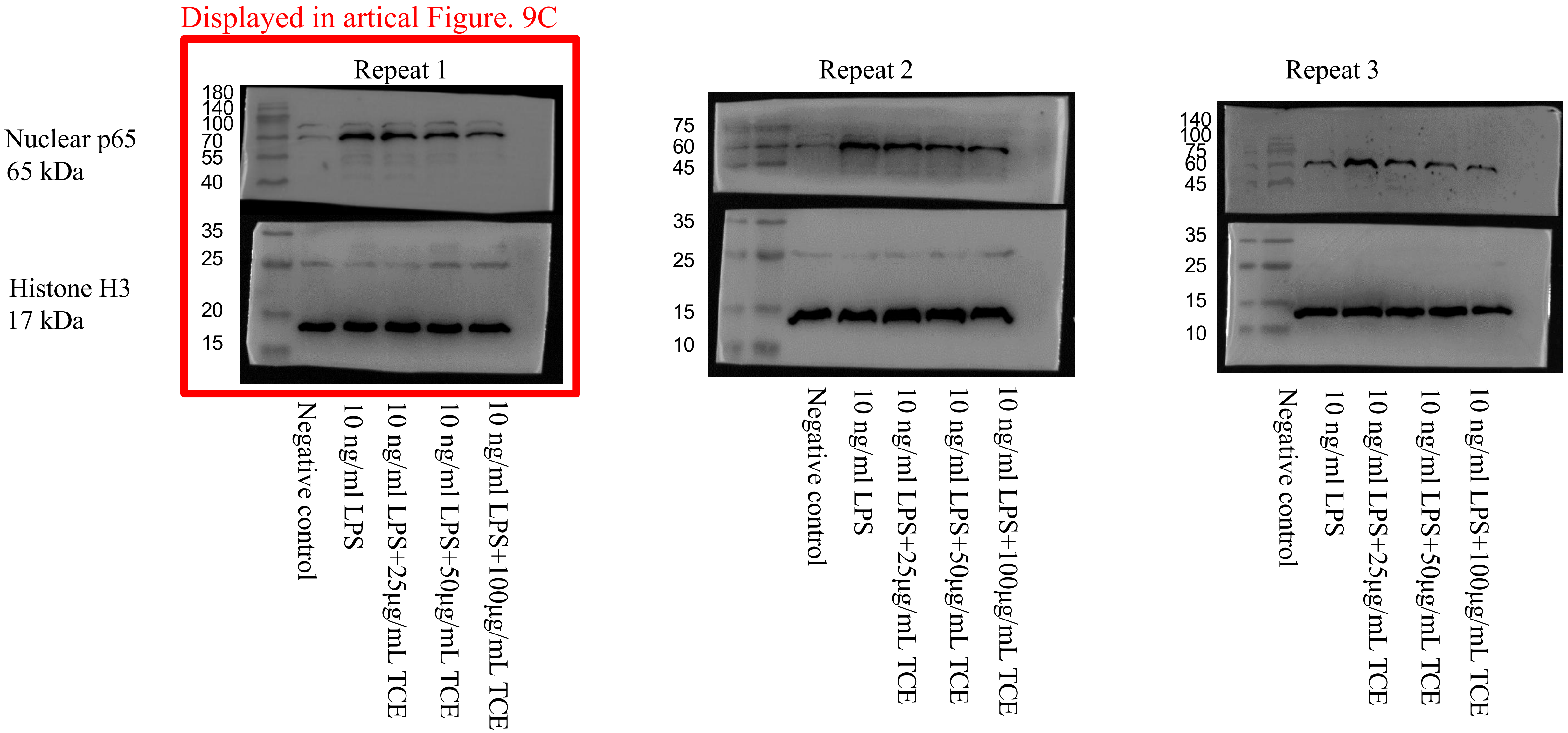

Figure. 10B p-ERK

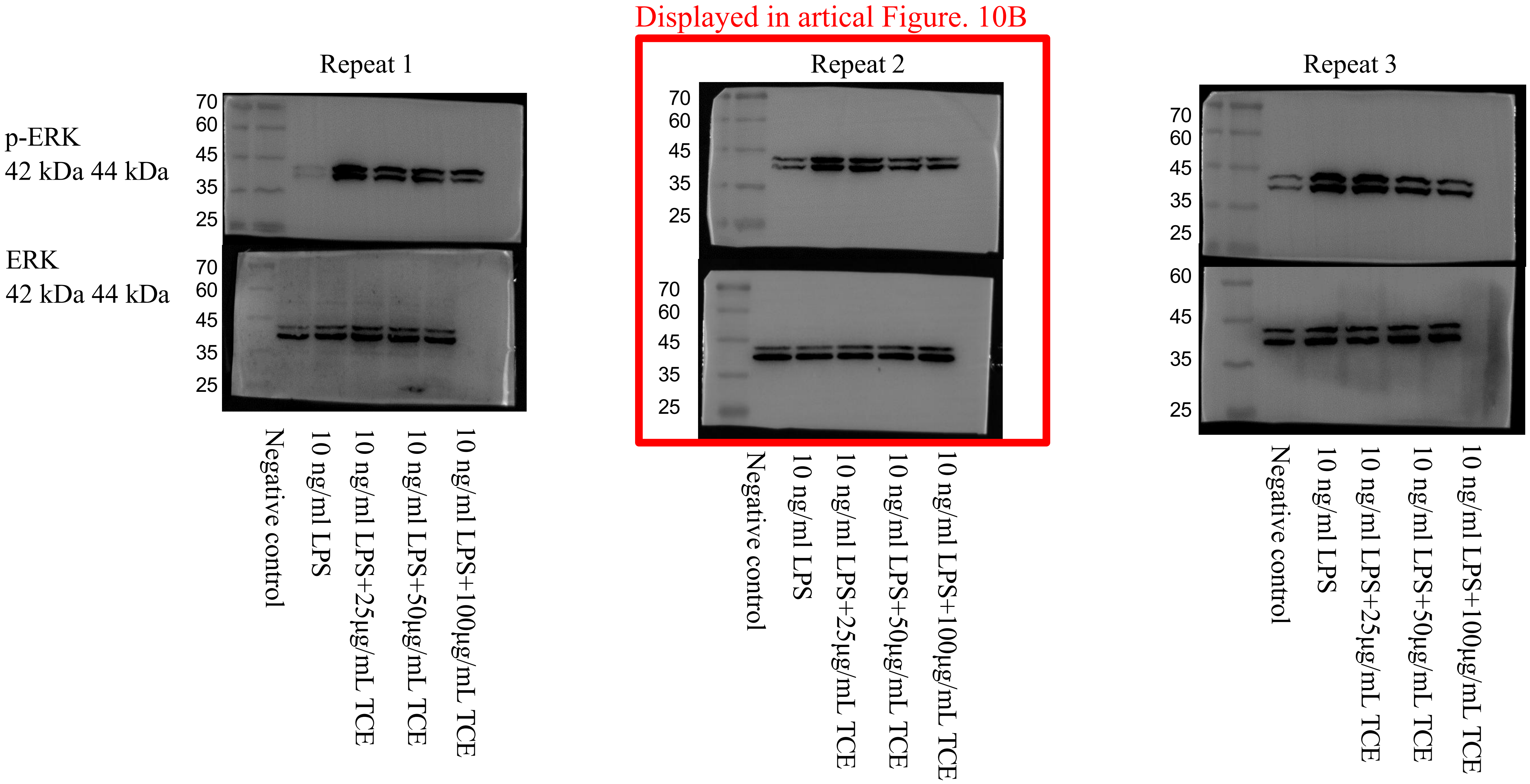

Figure. 10C p-JNK

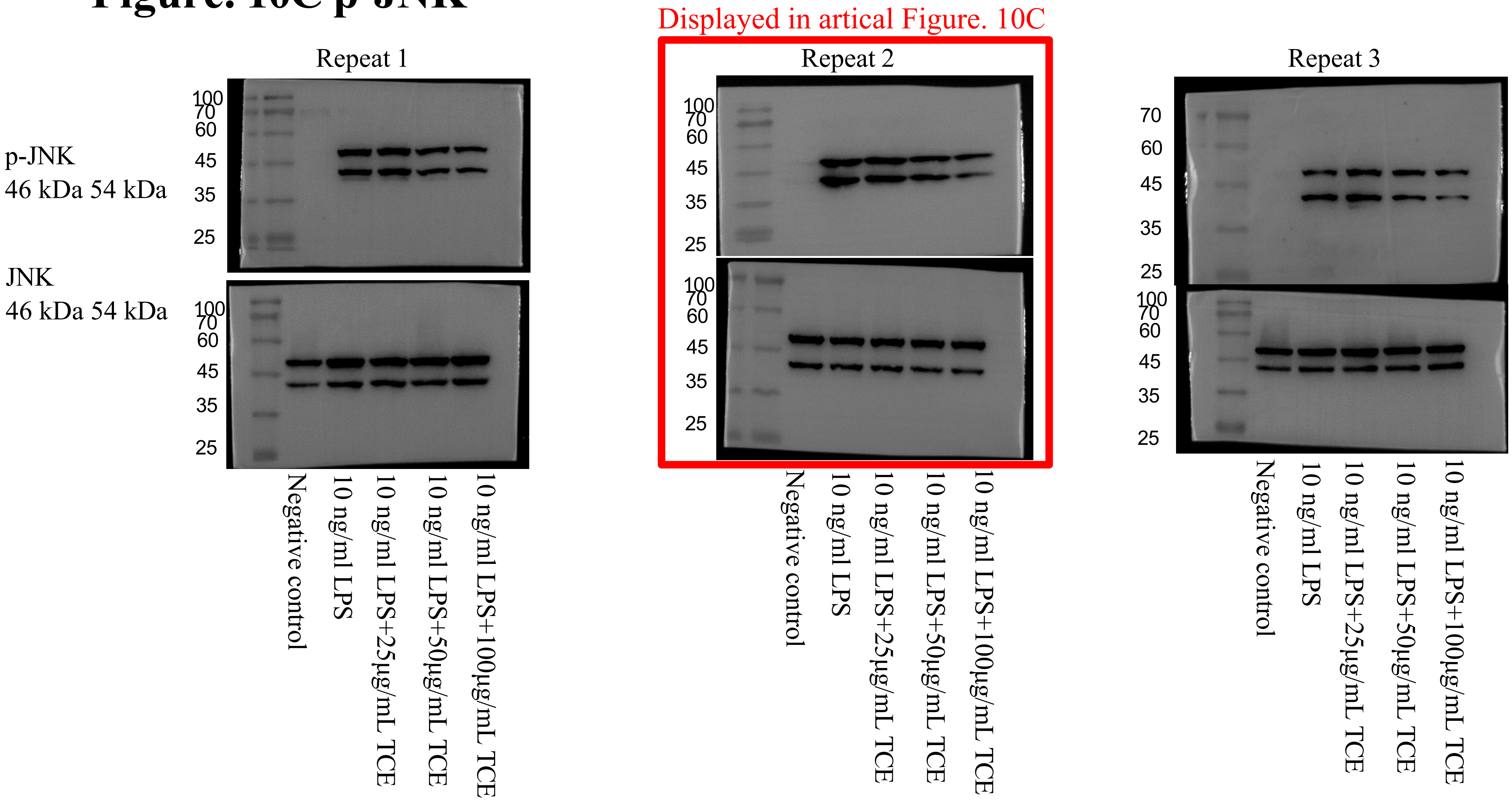

Figure. 10D p-p38

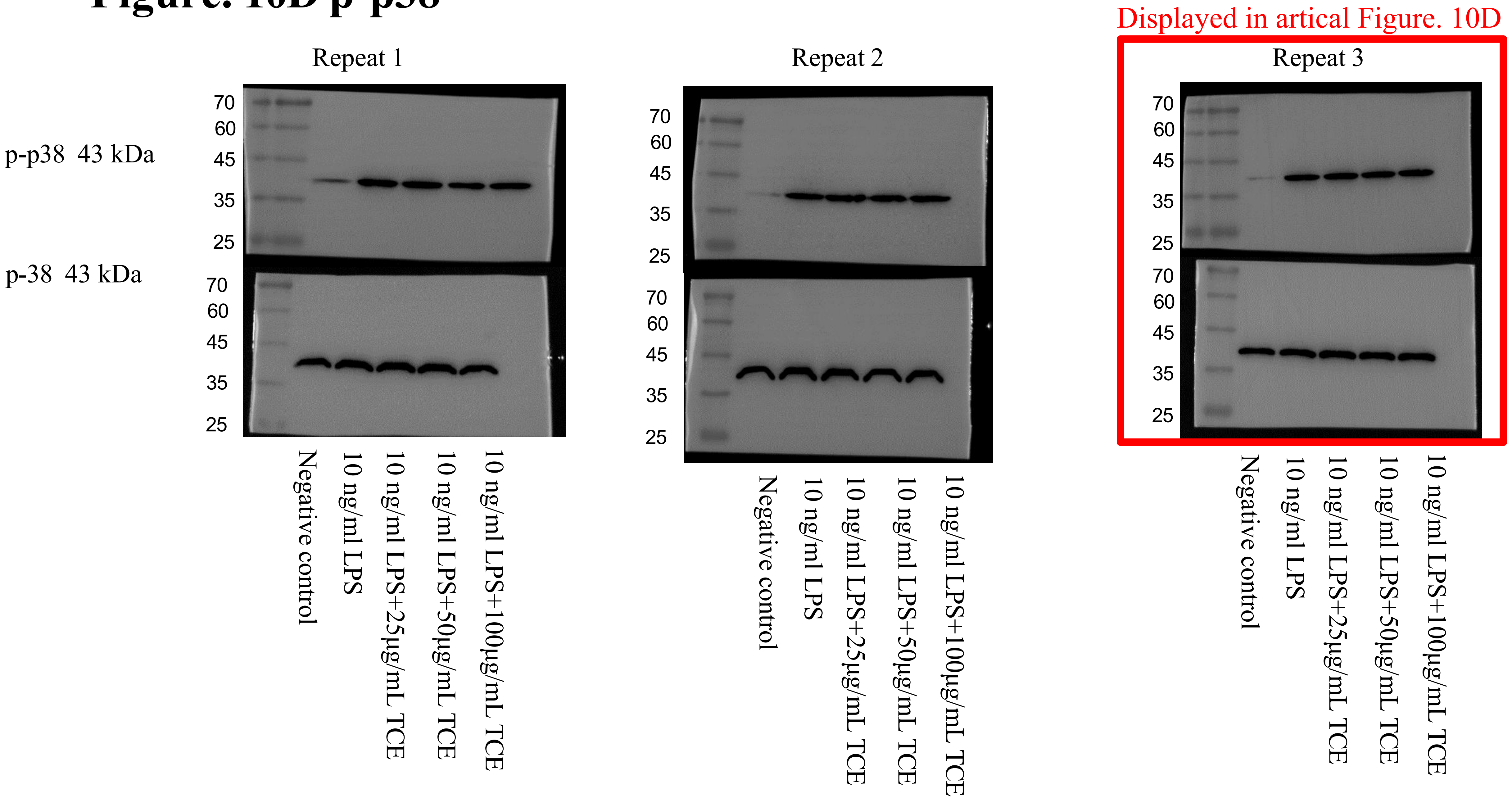

Supplement: Supplementary file 1 [file plants-15-02199-s001.zip › Raw wb images.pdf]
